# Description of a case of daily fever, developmental abnormalities, and a heterozygous M694I *MEFV* mutation

**DOI:** 10.1186/1546-0096-13-S1-P121

**Published:** 2015-09-28

**Authors:** G Pinto-Patarroyo, D Kastner

**Affiliations:** 1 National Institutes of Health, National Human Genome Research Institute, Bethesda, Maryland, USA

## Introduction

As a referral center for children with unexplained autoinflammatory disease, the NIH sees a spectrum of patients with previously unrecognized clinical syndromes. Here we present one such case.

## Objective

To describe a patient with unexplained fevers and a novel clinical presentation.

## Patients and methods

The patient is a 6 year-old boy referred from a small town in Mexico. The patient was screened for mutations in the known periodic fever syndrome genes by Sanger sequencing. Whole genome SNP genotyping and whole exome sequencing are under way.

## Results

The patient was referred to our clinic for evaluation of febrile episodes that started when he was 3 months old. Fever was present every day, and was associated with a rash that started in his feet and spread to his legs, with irritability, abdominal pain, diarrhea, arthralgia, and arthritis. During his first year of age, he was treated empirically for multiple infections, but continued to have daily fevers. As an infant, he underwent diagnostic testing for malignancy and infectious diseases that were negative. At 18 months of age he was diagnosed with Still's Disease. He underwent multiple treatments, including oral steroids, methotrexate, mycophenolate mofetil, etanercept and intravenous cyclophosphamide, none of which relieved his symptoms. When the patient was 3 years old he was started on tocilizumab; the fevers evolved from daily to episodic, occuring every 4 to 6 weeks and lasting 3 to 5 days. Genetic testing revealed a heterozygous M694I mutation in the *MEFV* gene. At age 5 the patient developed seizures. MRI of the brain showed hypo-intense lesions and for this reason tocilizumab was discontinued with an almost immediate return of his daily fevers and resolution of the seizures. At NIH, he was found to have growth and developmental delay, striking dysmorphic features including microcephalus, coarse facies, muscle hypotonia, and impressive joint hypermobility. He also displayed abnormal behavior. He was febrile, irritable, and had a papular rash in his feet and ankles; inflammatory markers were elevated. After he started anakinra 4mg/Kg once a day, his fevers, arthritis, and rash disappeared and his general status improved dramatically. Further genetic analysis is pending.

## Conclusions

At present it is unclear whether the patient's fevers and developmental abnormalities represent manifestations of a common genetic lesion, or are independent phenotypes. Although responsive to IL-1 inhibition, the fever syndrome appears atypical for FMF. Genomic analysis may help to resolve these issues.

## Consent to publish

Written informated consent for publication of their clinical details was obtained from the patient/parent/guardian/relative of the patient.

